# Explainable and Interpretable Model for the Early Detection of Brain Stroke Using Optimized Boosting Algorithms

**DOI:** 10.3390/diagnostics14222514

**Published:** 2024-11-09

**Authors:** Yogita Dubey, Yashraj Tarte, Nikhil Talatule, Khushal Damahe, Prachi Palsodkar, Punit Fulzele

**Affiliations:** 1 Department of Electronics and Telecommunication, Yeshwantrao Chavan College of Engineering, Nagpur 441110, India; mr.yashrajtarte@gmail.com (Y.T.); nikhiltalatule10@gmail.com (N.T.); khushaldamahe@gmail.com (K.D.); 2 Department of Electronics Engineering, Yeshwantrao Chavan College of Engineering, Nagpur 441110, India; prachi.palsodkar@gmail.com; 3 Directorate of Research and Innovation, SPDC, Datta Meghe Institute of Higher Education & Research, Wardha 442001, India; punitr007@gmail.com

**Keywords:** stroke prediction, machine learning, explainable AI, LIME, SHAP

## Abstract

**Background/Objectives:** Stroke stands as a prominent global health issue, causing con-siderable mortality and debilitation. It arises when cerebral blood flow is compromised, leading to irreversible brain cell damage or death. Leveraging the power of machine learning, this paper presents a systematic approach to predict stroke patient survival based on a comprehensive set of factors. These factors include demographic attributes, medical history, lifestyle elements, and physiological metrics. **Method:** An effective random sampling method is proposed to handle the highly biased data of stroke. The stroke pre-diction using optimized boosting machine learning algorithms is supported with explainable AI using LIME and SHAP. This enables the models to discern intricate data patterns and establish correlations between selected features and patient survival. **Results:** The performance of three boosting algorithms is studied for stroke prediction, which include Gradient Boosting (GB), AdaBoost (ADB), and XGBoost (XGB) with XGB achieved the best outcome overall with a training accuracy of 96.97% and testing accuracy of 92.13%. **Conclusions:** Through this approach, the study seeks to uncover actionable insights to guide healthcare practitioners in devising personalized treatment strategies for stroke patients.

## 1. Introduction

A brain stroke is one of the most severe diseases in which a blood clot or bleeding occurs in the brain during a stroke, which can result in long-term damage to the brain. This can affect a person’s movement, thinking, vision, or communication. It occurs when the body part that the blood-starved brain cells regulate ceases to function. Strokes are classified into two types: ischemic stroke and hemorrhagic stroke. In an ischemic stroke, there is a blood clot in the brain that passes through the patient’s bloodstream and becomes lodged. The second type of stroke—that is, hemorrhagic stroke—occurs in the brain when there is a leak of blood or rupture in the artery [1]. Therefore, the timely identification of a stroke is a must, and this can be performed by taking into consideration various parameters like gender, age, hypertension, heart disease, blood pressure, ever being married, work type, residential type, average glucose level, BMI, smoking status, etc. [2].

As per the World Health Organization (WHO) report, non-communicable illnesses accounted for 74% of deaths globally in 2019, with stroke being the second leading cause of death, affecting over 16 million people and accounting for around 11% of deaths [3]. It is a neurological condition that can develop from ischemia or hemorrhage of the brain arteries and often leads to diverse motor and cognitive deficits like movement, thinking, vision, or communication [4]. According to studies, four major areas of stroke problems have been identified for investigation, including prevention, diagnosis, therapy, and prognostication [5]. Stroke prognostication is the process of analyzing prospective outcomes and forecasting the future course of a stroke based on a variety of characteristics such as the severity of the stroke, the individual’s medical history, and the success of therapy.

Machine-learning (ML) approaches are increasingly being explored for stroke prognosis due to their ability to analyze large datasets and find complicated patterns those standard statistical methods may not identify [6]. Various ML methodologies are applied in the literature for stroke analysis like logistic regression (LR) [7,8,9,10,11], Naïve Bayes (NB) [12,13], support vector machine (SVM) [14,15], decision tree (DT), K-nearest neighbor (KNN), artificial neural network (ANN), boosting, bagging, random forest (RF), density-based clustering (DBSCAN), stacking, fuzzy clustering, ensemble-based methods [16,17,18], [19], and voting classifiers [20,21,22]. These algorithms have addressed the different research questions like stroke problems, features, or parameters, preprocessing and augmentation techniques, performance metrics, and clinical implications.

Ensemble learning has been shown to be the most effective classifier out of 179 different classifiers reported in the literature [16]. In ML, an ensemble is an assortment of trained models whose combined prediction is intended to improve the predictive performance of a single model with highly effective forecasting approaches with a large capacity for generalization without ignoring localized or specialized information [23]. XGBoost, LightGBM, CatBoost, RF, gradient boosting (GB), and AdaBoost are boosting algorithms that prioritize both speed and accuracy [24]. These boosting algorithms are gaining popularity because of their adaptability, resilience, and effectiveness in a variety of machine-learning applications, such as classification, regression, and recommendation systems. Furthermore, their efficient implementations offer rapid training and prediction timeframes, making them suitable for both small and large-scale applications [24,25].

The selection of the features for healthcare applications is a challenging task. Choosing the right feature set can increase learning accuracy, readability, and understanding by removing redundant features. Explainable Artificial Intelligence (XAI) focuses on building faith in AI systems by explaining their ML models when deployed on critical applications like healthcare. It delivers a set of ML approaches that allow human users to understand, trust, and develop more explainable models [26,27]. Various feature-based methods exist, including permutation feature importance, Partial Dependence Plots (PDPs), Individual Conditional Expectation (ICE) plots, Accumulated Local Effects (ALEs) plots, Global Surrogate Models, Local Interpretable Model-agnostic Explanations (LIMEs), and Shapley Additive Explanations (SHAP), each with their own merits and limitations [25]. Each interpretability approach has its place, based on the task’s unique requirements and restrictions. Understanding their strengths and limits might assist practitioners in selecting the best approach for their interpreting needs [28,29].

In many of the methods described above, data imbalance was not considered which results in overfitting and an explanation of the prediction was also not discussed. This paper presents an optimized boosting machine-learning approach for stroke prediction that involves hyperparameters’ tuning and model-agnostic explanations. We handled the challenge of highly imbalanced stroke data by employing strategic random sampling approaches, especially downsampling and regenerative sampling. Our methodology integrates demographic attributes, medical history, lifestyle factors, and physiological metrics for comprehensive stroke prediction analysis. We use rigorous statistical analysis to evaluate the value of each variable contributing to stroke risk and optimize feature selection to improve prediction accuracy. Furthermore, we employ model-agnostic explanation techniques like SHAP and LIME to deliver extensive insights to medical practitioners, allowing them to make better decisions. Our technique provides a strong framework for reliable stroke prediction while also giving interpretable reasons for improved understanding and trust in predictive algorithms. The major contributions of this paper are as follows:

- To propose effective random sampling to address the issue of highly imbalanced stroke data using downsampling and regenerative sampling methods.
- Optimized boosting machine learning with hyperparameter tuning for stroke prediction using analysis of demographic attributes, medical history, lifestyle elements, and physiological metrics.
- Systematic statistical analysis to check the importance of each feature contributing to stroke and optimizing features for stroke prediction with better accuracy.
- Model agnostic-based explanation using SHAP and LIME for stroke prediction to provide an insight into decision-making for medical professionals.

**Figure 1:**
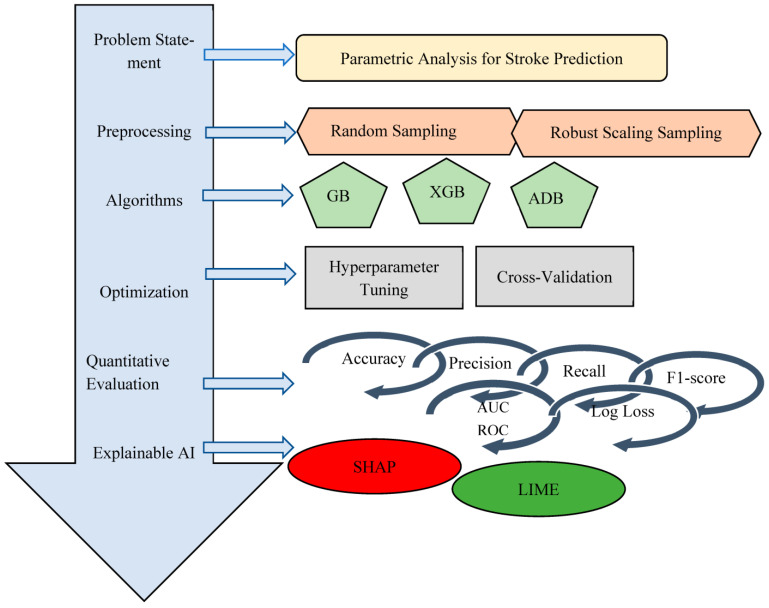
Proposed framework for stroke prediction using boosting algorithms with LIME and SHAP explanation.

A combination of downsampling and upsampling provides a proper framework for imbalance data with a better accuracy of stroke prediction. Feature analysis gives an insight for stroke prediction using fewer features. And finally, the explanation of these results on stroke prediction is supported by LIME and SHAP. The rest of the paper is organized as follows. Section 2 describes the material and methods used for stroke prediction with parametric analysis and the proposed framework. Section 3 and Section 4 includes results and discussion with quantitative evaluation and explanation using LIME and SHAP. Section 5 concludes this research work. Figure 1 describes the complete framework employed for stroke prediction using boosting algorithms with LIME and SHAP explanation.

## 2. Material and Methods

In this section, we present a dataset description with explorative data analysis and data preprocessing techniques.

### 2.1. Dataset Description

The prediction of brain stroke is based on the Kaggle dataset [30] accessed in September 2024. The dataset contains information from a sample of individuals, including both stroke and non-stroke cases. There are a total of 4981 samples. The database is biased toward the negative class. Out of 4981 samples, 4733 patients were not having a brain stroke and 248 patients were suffering from a brain stroke. The data contain 2074 women patients’ data and 2907 male patients’ data. Out of 2074 female data, 108 women were suffering from a brain stroke and 1966 patients were not having a brain stroke. Similarly, out of 2907 male data, 140 patients were suffering from a brain stroke and 2767 patients were not having a brain stroke. The distribution of the complete data is detailed in Table 1.

It can be observed from Table 1 that the original dataset is highly imbalanced. If this biased dataset is used for predictive modeling, the problem of overfitting will arise or we may not obtain proper results.

The prediction of a stroke is based on 10 features from the dataset. These are age, gender, hypertension, having heart disease or not, being married or unmarried, work type, residence type, average glucose level, body mass index, and smoking status. Out of these 10 features, 3 features have numerical values. These features are detailed in Table 2.

It can be observed from Table 2 that the average age of individuals from the dataset ranging from 8 years to 82 years, with an average of 43 years, in which age offers a diverse spectrum of values. Advanced age has been consistently associated with an elevated risk of stroke, making this numerical representation essential for uncovering age-related patterns and their impact on stroke prediction. It can be observed from Table 2 that the average age of individuals who have suffered a stroke is 68 years, while individuals without a stroke have an average age of 42 years. This disparity underscores the significant influence of age as a contributing factor to stroke susceptibility. Another numerical parameter of significance is the average glucose level in the blood. Ranging from a minimum of 55.1 mg/dL to a maximum of 271.7 mg/dL, with an average of 105.4 mg/dL, this parameter provides insights into the potential influence of glucose levels on stroke occurrence. Elevated blood glucose levels have been linked to vascular issues, underscoring the importance of quantifying this factor in stroke prediction. It can be observed from Table 2 that the average blood glucose level among stroke-affected individuals is 132.2 mg/dL, whereas individuals without a stroke exhibit an average glucose level of 104.6 mg/dL. Elevated blood glucose levels have been associated with vascular complications, highlighting the potential role of glucose regulation in stroke risk.

The average body mass index (BMI) of individuals, ranging from 14.0 to 48.9, with an average of 28.5, offers valuable insights into the relationship between body composition and stroke risk. High BMI values are associated with an increased likelihood of stroke, making this numerical representation instrumental in identifying correlations between BMI and stroke incidence. Integrating these numerical parameters into the stroke prediction framework enhances the accuracy of risk assessment. By quantifying age, average glucose levels, and BMI, healthcare professionals gain a more comprehensive understanding of individual stroke risk profiles, enabling them to tailor treatment strategies and interventions effectively. Individuals who have experienced a stroke possess an average body mass index (BMI) of 30.2, whereas those without stroke exhibit an average BMI of 28.4. The higher BMI among stroke cases emphasizes the potential impact of body composition on stroke susceptibility.

These numerical contrasts provide valuable insights into the distinctive characteristics of individuals with and without stroke. The higher average age, glucose levels, and BMI among stroke cases underscore the importance of these parameters as potential predictors of stroke occurrence. Such insights are instrumental in refining stroke prediction models and guiding healthcare practitioners in targeted interventions for stroke prevention and management. Out of 10 features, 7 features have categorical values. These 7 features are detailed in Table 3.

Gender, reflecting the binary distinction between female (0) and male (1), is an essential parameter to consider. Gender-related factors may contribute to stroke risk variations, necessitating its inclusion in the prediction framework. Hypertension evaluates the presence of hypertension among individuals. It is represented by binary values—0 for “No” and 1 for “Yes”. Hypertension, if present, has been identified as a significant risk factor for stroke, making this categorical assessment pivotal in assessing stroke vulnerability. The presence of heart disease is indicated by values of 0 for “No” and 1 for “Yes”. Heart disease is closely linked to stroke risk, emphasizing the importance of this parameter in determining potential stroke occurrences.

Ever being married is represented as 0 for “No” and 1 for “Yes”. This parameter captures the marital status of individuals. Marital status can influence lifestyle and support networks, potentially impacting stroke risk, making it a noteworthy factor for stroke prediction. Work type classifies individuals based on their work engagement. The categorical attributes include 0 for private, 1 for self-employed, 2 for government job, and 3 for never worked. Occupational factors can impact lifestyle choices and stress levels, making work type an influential determinant of stroke risk. Residence type distinguishes between urban (0) and rural (1) residences; this parameter highlights the potential impact of living environments on stroke susceptibility. Access to healthcare resources and lifestyle differences in different residence types can contribute to varying stroke risks.

By considering these categorical parameters and their associated attributes, the stroke prediction model gains a comprehensive understanding of individuals’ characteristics and their role in influencing stroke risk. This holistic approach facilitates informed decision-making for healthcare practitioners, enabling tailored treatment strategies and interventions to mitigate stroke susceptibility.

### 2.2. Explorative Data Analysis of Features

The features can be categorized as demographic features, physiological metrics, medical history, and lifestyle elements. The effect of these features on the possibility of stroke is described below.

#### 2.2.1. Demographic Features

Age and gender are the demographic characteristics which have an influence on the outcome of stroke prediction.

**Figure 2:**
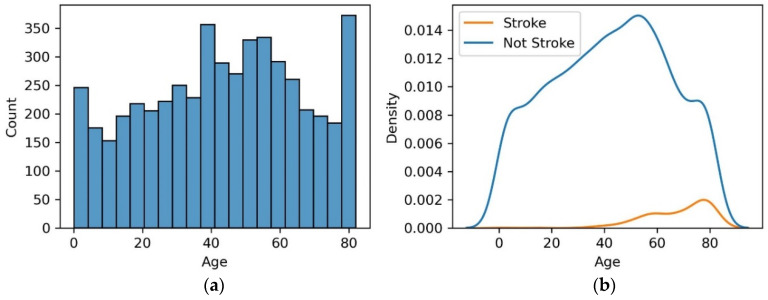
(**a**) Histogram plot and (**b**) probability density function of age with respect to stroke.

Age: Age is a substantial factor in stroke prediction, revealing a nuanced relationship with stroke survival. The histogram plot and probability density function of age with respect to stroke is shown in Figure 2.

From Figure 2, it is observed that advanced age exhibits a nonlinear trend, indicating that while age is a risk factor, its impact is influenced by various other variables. As individuals grow older, their risk of experiencing adverse stroke outcomes increases. This highlights the importance of tailoring medical interventions and preventive strategies to address the unique needs of elderly stroke patients. Healthcare professionals should consider age as a critical factor when assessing stroke risk and designing personalized treatment plans.

**Figure 3:**
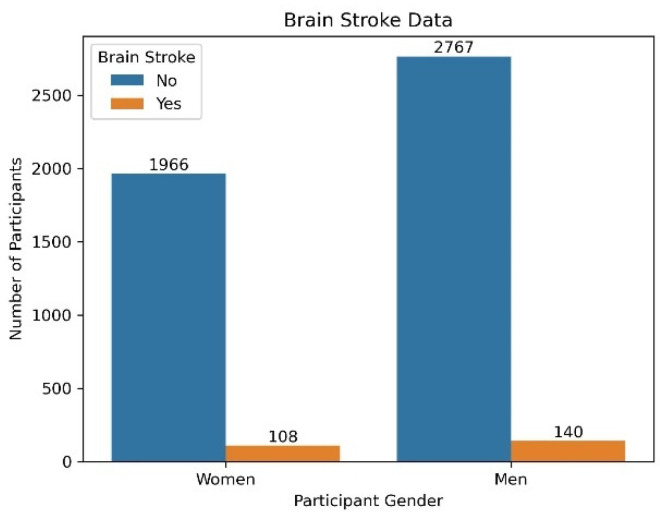
Gender-wise distribution of stroke data.

Gender: Gender-related factors play a role in understanding stroke vulnerability. The analysis suggests that there are gender-related differences in stroke incidence. This highlights the need for gender-sensitive approaches in stroke prevention and treatment strategies. Healthcare practitioners should consider gender-specific risk factors and tailor interventions accordingly. By acknowledging gender-related nuances, healthcare providers can develop more effective and targeted approaches to mitigate stroke risk among both male and female individuals. From Figure 3, it is observed that out of 248 patients with stroke, 140 (56.45%) male patients were prone to stroke and 108 female patients (43.5%) were susceptible to stroke.

#### 2.2.2. Physiological Metrics

**Figure 4:**
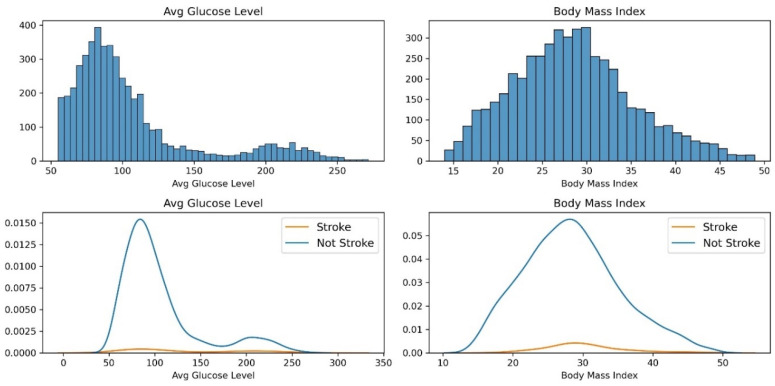
Histogram plot and probability density function of average glucose level and body mass index.

Average glucose levels and body mass index are physiological metrics. The histogram plot and probability density function for these features concerning strokes is shown in Figure 4.

The average blood glucose level offers insights into the stroke risk by highlighting the importance of glycemic control. Elevated glucose levels are associated with an increased risk of stroke, as they can impact blood vessels and contribute to vascular damage. Managing blood sugar levels through lifestyle modifications and medical interventions becomes pivotal in stroke prevention. Healthcare providers should focus on educating patients about the link between glucose control and stroke risk and emphasize the importance of adopting healthy dietary habits and monitoring blood sugar levels. BMI serves as a valuable indicator of body composition and obesity-related stroke risk. The study indicates that individuals with higher BMIs face an increased likelihood of strokes. This underscores the role of weight management and healthy lifestyle practices in reducing stroke susceptibility. Healthcare professionals should prioritize educating patients about the importance of maintaining a healthy weight, engaging in regular physical activity, and making dietary choices that promote optimal BMI. By addressing obesity as a modifiable risk factor, healthcare providers can contribute significantly to stroke prevention.

#### 2.2.3. Medical History

Hypertension and heart disease are the medical history factors from the dataset for predicting stroke. The effect of these parameters on the outcome of stroke is described below.

Hypertension: Hypertension emerges as a significant contributor to stroke risk. The study reveals that individuals with high blood pressure are at an elevated risk of experiencing a stroke. The intricate relationship between hypertension and stroke underscores the necessity of effective blood pressure management as a vital strategy for stroke prevention. Healthcare practitioners should prioritize regular blood pressure monitoring, lifestyle modifications, and appropriate medications to mitigate hypertension-related stroke risk. By addressing this primary risk factor, healthcare professionals can make substantial strides in reducing the incidence of strokes.

From Table 4, the observed data provide useful insights into the association between hypertension and the chance of developing a stroke in the analysis of the “hypertension” characteristic concerning stroke status. A stroke was observed in roughly 13.78% (66 cases) of adults with hypertension (479). The vast majority of patients, 86.22% (413), are not expected to suffer a stroke. In contrast, among those without hypertension (4502), around 4.05% (182 patients) are predicted to have a stroke. The vast majority of patients, 95.95% (4320), are not expected to suffer a stroke. This study found a link between hypertension and the chance of having a stroke. Individuals with hypertension have a much greater rate of stroke cases (13.78%) than those who do not have hypertension (4.05%). This emphasizes the significance of hypertension management as a critical component in stroke prevention.

Heart Disease: The presence of heart disease showcases a strong association with stroke prognosis. Individuals with a history of heart disease are more susceptible to experiencing strokes. This emphasizes the interconnectedness of cardiovascular health and stroke risk. Cardiac care and preventive measures are crucial in reducing the likelihood of stroke among individuals with heart disease. Healthcare providers should adopt a comprehensive approach that includes managing heart conditions and addressing stroke risk factors to optimize patient outcomes and overall cardiovascular health.

From Table 5, it can be observed that, out of 275 patients having heart disease, 47 patients (about 17.09%) have a chance of having a stroke and 228 i.e., 82.9% of patients will not have a stroke. Similarly, out of 4706 patients, 201 patients (about 4.2%) may have a stroke and 4505, i.e., 95.72% of patients, will not have a stroke.

#### 2.2.4. Lifestyle Elements

Marital status, work type, residence type, and smoking status are the four lifestyle elements from the dataset which has influence on the prediction of stroke.

Marital Status (Ever Married): Marital status emerges as an intriguing sociodemographic factor that can influence stroke outcomes. The study suggests that individuals who have been married may exhibit a more favorable prognosis. This could be attributed to the potential social and support network benefits that married individuals may enjoy. Healthcare professionals should recognize the significance of social support in stroke recovery and consider incorporating measures to enhance support systems for unmarried patients. By addressing the emotional and psychological aspects of stroke rehabilitation, healthcare practitioners can contribute to improved patient outcomes. 

From Table 6, it is observed that, patients who were married had a total count of 3280 in the study of the “Ever Married” characteristic relating to stroke status. In total, 6.68% (219 patients) of these people were recognized as having had a stroke. Most patients, 93.32% (3061), are not expected to suffer a stroke. Patients who were not married, on the other hand, had a total count of 1701. Approximately 1.70% (29 patients) of these people were said to be at risk of having a stroke. The vast majority, or 98.3% (1672 patients), are not expected to suffer a stroke.

This investigation demonstrates that among patients with stroke, those who were married had a greater risk of having a stroke than those who were not married. Approximately 6.68% of married patients are at risk of having a stroke, but only 1.70% of unmarried patients are at risk. However, most of both groups are not predicted to experience a stroke.

Work Type: Work type provides insights into the potential influence of occupational factors on stroke outcomes. The study reveals varying stroke risks associated with different work types, reflecting the impact of occupational environments on individual health and well-being. Healthcare professionals should collaborate with occupational health experts to identify workplace-related risk factors and implement preventive measures. By addressing occupational influences on stroke risk, healthcare providers can contribute to creating safer work environments that promote employee well-being and reduce the incidence of strokes.

From Table 7, it is observed that, patients with a government job had a total count of 673 in the study of the “work type” aspect affecting stroke status. A total of 0.3% (2 people) were recognized as having had a stroke. Many patients, 99.7% (671), are not expected to develop a stroke. Individuals having a private job numbered 2860 in total. Out of these individuals, 5.17% (148 patients) are predicted to have a stroke, whereas the vast majority, consisting of 94.83% (2712 patients), are not expected to have a stroke. There were a total of 804 self-employed people. Approximately 8.08% (65 patients) of these individuals were recognized as having had a stroke, whereas the vast majority, 91.92% (739 patients), are not expected to have a stroke. In addition, those looking after children totaled 644. In total, 5.12% (33 patients) of these people are predicted to have a stroke, whereas the vast majority, 94.88% (611 patients), are not expected to have one. This study found that different occupational positions have distinct degrees of stroke prevalence. Individuals with private occupations or who are self-employed have greater rates of stroke than those with government positions or children.

Residence Type: Residence type, whether urban or rural, offers valuable insights into stroke risk by highlighting potential disparities in healthcare access and environmental factors. Individuals residing in different settings may have distinct stroke risk profiles based on the availability of healthcare resources and exposure to environmental triggers. Healthcare practitioners should consider residence type when assessing stroke risk and tailor preventive strategies accordingly. By addressing the unique challenges associated with urban and rural environments, healthcare providers can contribute to reducing stroke risk and improving patient outcomes. The data shed light on the relationship between the place of residence and the chance of developing a stroke in the examination of the “residence type” characteristic concerning stroke status. 

Table 8 shows that, with a total population of 2532 people living in rural regions, roughly 5.33% (135 patients) were recognized as having had a stroke. Many patients, 94.67% (2397), are not expected to suffer a stroke. In contrast, among the 2449 people living in urban regions, around 4.61% (113 patients) are predicted to have a stroke and 95.84% (2336 patients) are not expected to suffer a stroke. This study found a complex link between dwelling type and the chance of having a stroke. While persons in rural regions have a slightly greater rate of stroke cases (5.33%) than those in urban areas (4.61%), it is important to highlight that many people in both categories are not likely to suffer a stroke.

Smoking Status: Smoking status emerges as a critical determinant in stroke prediction. The study underscores the well-established link between smoking and stroke risk, with different smoking categories showcasing varying levels of susceptibility. Healthcare professionals should prioritize smoking cessation interventions as a fundamental strategy for reducing stroke risk. By offering smoking cessation support, counseling, and resources, healthcare providers can empower individuals to quit smoking and thereby significantly reduce their chances of experiencing a stroke. The data provide useful insights into the relationship between smoking behaviors and the chance of developing a stroke in the evaluation of the “smoking status” feature about stroke status. 

Table 9 shows that, a stroke was discovered in roughly 3.13% (47 cases) of those with unknown smoking status, totaling 1500. The vast majority, or 96.87% (1453 individuals), are not expected to suffer a stroke. Approximately 4.84% (89 patients) of those who have never smoked (1838) are at risk of having a stroke and 95.16% (1749 patients) are not expected to suffer a stroke. Former smokers (867) have a somewhat greater rate, about 8.07% (70 patients), of having had a stroke, and 91.93% (797 patients) are not expected to suffer a stroke. In the group of current smokers (776), roughly 5.41% (42 individuals) are predicted to have a stroke and 94.59% (734 patients) are not expected to develop a stroke. This study sheds light on the relationship between smoking status and the chance of having a stroke. Individuals with uncertain smoking status and past smokers had a somewhat greater incidence of stroke cases than never-smokers and present smokers. However, it is critical to understand that most people in these groups are not likely to suffer a stroke

### 2.3. Feature Contribution

To find the importance of each feature in the prediction of strokes, the correlation is calculated using a correlation coefficient. The correlation coefficient r between the input variable $x_{i}$ with the output variable $y_{i}$ is calculated using

(1) $$r=\frac{\sum_{i=1}^{N}\left(x_{i}-\bar{x}\right)\left(y_{i}-\bar{y}\right)}{\sqrt{\sum_{i=1}^{N}{\left(x_{i}-\bar{x}\right)}^{2}\sum_{i=1}^{N}{\left(y_{i}-\bar{y}\right)}^{2}}}$$

Here, $\bar{x}$ is the mean of input variable, $\bar{y}$ is the mean of output variable, and N is the total number of samples. The correlation coefficient values obtained for each feature with respect to the output variable stroke are shown in Table 10.

From Table 10, it can be observed that a positive correlation (0.246) exists between age and the likelihood of a stroke. As age increases, the chance of a stroke also tends to increase. Heart disease (0.134), average glucose level (0.133), hypertension (0.131), ever being married (0.108), and work type (0.062) also show a positive correlation with stroke, suggesting that individuals with these characteristics may be more likely to experience a proficient efficient. Residence type (−0.016), body mass index (0.056), and smoking status (0.054793) exhibit relatively weak correlations with stroke, indicating a less clear relationship, whereas gender (0.0088) appears to have a negligible correlation with stroke.

### 2.4. Data Preprocessing

**Figure 5:**
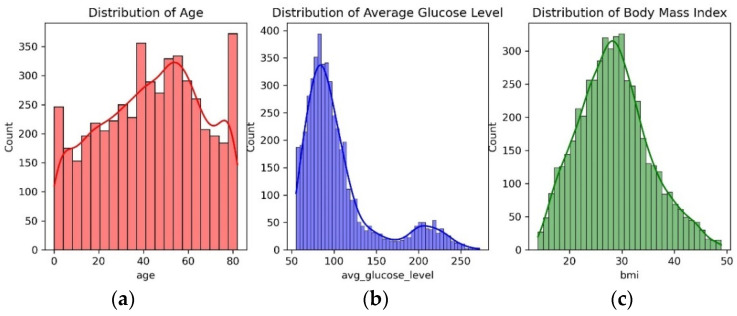
Distribution of features: (**a**) Age; (**b**) Average glucose level; (**c**) Body mass index for stroke prediction.

Out of the 10 features from the dataset, only age, average glucose level, and body mass index have numerical values with distributions as shown in Figure 5.

It can be observed from Figure 5 that the feature age and average glucose levels are not normally distributed. And the range of age varies from 8 to 80, the range of average glucose level is from 55 to 270, and the range of body mass index is from 14 to 50. With this feature distribution, the modeling may be biased towards the average glucose level. Hence, robust scaling is used to bring these features to the same scale and to remove outliers if any. The robust scaling is performed as per the Equation (2):

(2) $$X^{\prime }=\frac{X-Q_{2}}{Q_{3}-Q_{1}}$$

Here, X is the feature value before scaling, $X^{\prime }$ is the feature value after scaling, $Q_{2}$ is the median (50th percentile) value of the feature, $Q_{3}$ is the 75th percentile value of the feature, and $Q_{1}$ is the 25th percentile value of the feature. The other three features of heart disease, hypertension, and ever being married are categorical features; hence, no scaling is used for those features.

### 2.5. Random Sampling

**Figure 6:**
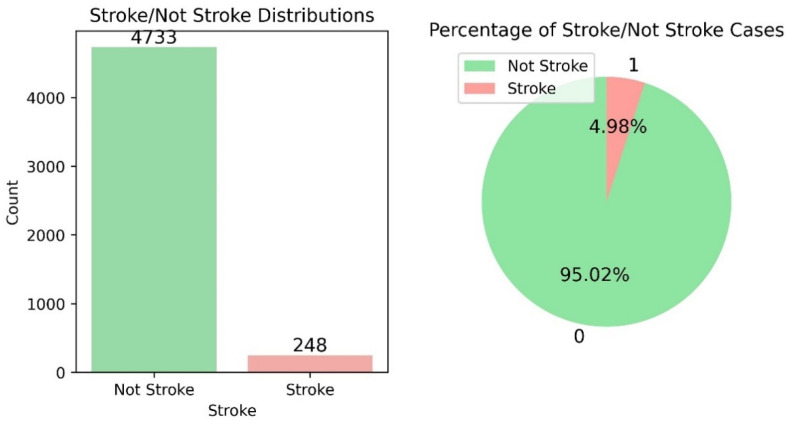
Distribution of patients’ data in the stroke and not-stroke categories.

The dataset used for the stroke prediction is biased toward the negative class (4733 out of 4981), which is far greater than the samples for the positive class (248 out of 4981). About 4.98% of the dataset represents of patients with a stroke and 95.02% of the dataset represents patients with no stroke. Figure 6 shows the distribution of patient’s data in the stroke and not-stroke categories. 

This biased dataset will result in overfitting. To avoid this, data sampling is used before modeling. For the best performance, a combination of under-sampling and over-sampling is proposed. Firstly, the not stroke is downsampled to the count of stroke samples, i.e., 248 samples for both stroke and not stroke. After that, upsampling is performed in this new dataset using a regenerative technique for stroke data.

There are a total 4733 not-stroke samples and 248 stroke samples; hence, downsampling is performed on the not-stroke samples with a sampling rate of $\frac{248}{4733}=0.52=50\%$. With random sampling, the chances of selection of the data sample from the not-stroke data only once will be $P=\frac{n}{N}=\frac{2480}{4733}=50\%$. Similarly, the chances of selection of the data samples from the not-stroke data more than once will be $P=1-{\left(1-\left(\frac{1}{N}\right)\right)}^{n}=1-{\left(\frac{N-1}{N}\right)}^{n}=1-{\left(\frac{4732}{4733}\right)}^{2480}=1-0.999^{2480}=0.916$.

The dataset distribution after performing downsampling is shown in Table 11.

After downsampling, upsampling is performed on samples with stroke cases. These 248 samples are increased with a sampling rate of 10%. This results in an equal number of samples for stroke and not-stroke cases as given in Table 12.

### 2.6. Modeling by Machine-Learning Algorithms

After data preprocessing, the machine-learning algorithms are applied on this dataset for predictive modeling of stroke disease using very few features. The boosting ML algorithms are applied to create the model for stroke prediction using selected features which are age, hypertension, average glucose level, heart disease, and ever being married. The boosting algorithms are discussed below.

#### 2.6.1. Gradient Boosting (GB)

Gradient boosting [31,32] is a machine-learning approach that optimizes a loss function by merging the predictions of numerous weak learners, typically decision trees. On each iteration, it trains models sequentially by fitting the negative gradient or pseudo-residuals of the loss function from prior models. The weight updation in gradient boosting is

(3) $${\hat{y}}_{m+1}={\hat{y}}_{m}+\alpha \times h_{m+1}\left(x\right)$$

where ${\hat{y}}_{m}$ is the forecast of the group of m trees, α is the learning rate, and $h_{m+1}\left(x\right)$ is the ${\left(m+1\right)}^{th}$ tree that minimizes the loss function. The loss function can be chosen based on the problem, such as exponential loss for classification or the mean squared error for regression [33].

Gradient boosting increases model accuracy and generalization by including regularization components in the loss function, such as tree complexity or L2 regularization. It also employs techniques such as shrinkage, pruning, parallelization, and distributed computing to improve the algorithm’s performance and efficiency.

#### 2.6.2. Extreme Gradient Boosting

XGBoost gradient boosting (XGB) [34] is a machine-learning technique that optimizes a loss function via gradient boosting by integrating the predictions of many weak learners. On each iteration, it trains models sequentially by fitting the negative gradient or pseudo-residuals of the loss function from prior models. The objective function of XGB is

Objective Function = Loss + Regularization

(4) $$Objective\;Function=\sum_{i=1}^{n}l\left(y_{i},{\hat{y}}_{i}\right)+\sum_{k=1}^{K}\Omega \left(f_{k}\right)\;=\sum_{i=1}^{n}l\left(y_{i},\sum_{k=1}^{K}f_{k}(x_{i})\right)+{\sum_{k=1}^{K}\gamma T_{k}+\frac{1}{2}\left\|w_{k}\right\|}^{2}$$

where l is a differentiable loss function, such as logistic loss for classification, $y_{i}$ is the true label, ${\hat{y}}_{i}$ is the predicted label, $f_{k}$ is the $k^{th}$ weak learner (decision tree), K is the number of trees, Ω is a regularization term, γ is a parameter for tree complexity, $T_{k}$ is the number of leaves in the $k^{th}$ tree, λ is a parameter for L2 regularization, and $w_{k}$ is the vector of scores on leaves of the $k^{th}$ tree.

The objective function is minimized by adding one tree at a time and utilizing gradient descent and Newton’s technique to obtain the ideal structure and leaf scores of each tree. The negative gradients, also known as pseudo-residuals, are computed:

(5) $$pseudo\_residuals=\nabla l\left(y,\hat{y}\right)$$

Here, ∇ is the gradient operator.

The next model tries to reduce these residuals by adding a weighted tree:

(6) $${\hat{y}}_{m+1}={\hat{y}}_{m}+\alpha h_{m+1}\left(x\right)$$

where the hyperparameter α is the learning rate and $h_{m+1}$ is the ${\left(m+1\right)}^{th}$ tree that minimizes the squared sum of pseudo-residuals. XGBoost additionally employs techniques such as shrinkage, pruning, parallelization, and distributed computing to increase the algorithm’s performance and efficiency. XGBoost improves gradient boosting by incorporating regularization terms to minimize overfitting, employing higher-order gradients to handle large-scale and complicated data.

#### 2.6.3. Adaptive Boosting

Adaptive boosting (ADB) [35,36] is a prominent supervised learning approach primarily tailored for tackling classification problems. Each instance within the training data is endowed with a weight, and ADB undertakes iterative rounds of training, meticulously progressively emphasizing misclassified examples. This iterative refinement fortifies the learning process, systematically steering the ensemble towards a refined predictive prowess. As these individual weak learners contribute their predictions, their significance is proportionately weighed based on their performance. Consequently, their collective insights culminate in the formulation of a final prediction, a synthesis of the ensemble’s accumulated knowledge. The weight α allocated to weak learners is calculated using

(7) $$\alpha =\frac{1}{2}\ln\left(\frac{1-\varepsilon }{\varepsilon }\right)$$

Here, ε is the weak learners’ weighted mistake rate. The updated weight of instance i at the t+1 iteration is calculated using the old weight w(t,i), actual label $y_{i}$.

(8) $$w\left(t+1,i\right)=w\left(t,i\right)\times \exp\left(\frac{-\alpha \times y_{i}\times h_{t}\left(x_{i}\right)}{z_{i}}\right)$$

Here, $h_{t}\left(x_{i}\right)$ is the weak learners’ prediction for instance i at the t iteration and $z_{t}=\sum w\left(t,i\right)$ is the normalization function. The loss function L of AdaBoost is calculated using

(9) $$L=\sum_{i=1}^{N}\exp\left(-y_{i}\times H\left(x_{i}\right)\right)$$

Here, $H\left(x_{i}\right)$ is ADB’s final prediction for the instance i and the total is calculated over all training examples. This loss function measures the extent of misclassification errors for each instance, assigning higher penalties to misclassified data points. As ADB aims to minimize this loss, subsequent weak learners prioritize correcting previous mistakes, collectively enhancing the model’s accuracy over iterations.

### 2.7. Explainable Artificial Intelligence (XAI) in Model Explanation

XAI gives precise and explainable justifications for its decisions and predictions. The main goal of XAI is to develop reliable and evident AI systems to understand the outcomes. XAI techniques found in machine learning can be described as model-based, post hoc, model-specific, model-agnostic, global, and local. These explanation methods can be categorized into three types: visual, textual, and example-based. Under visual-based techniques, we have backpropagation-based approaches and perturbation-based approaches. The SHAP technique falls into the category of backpropagation-based approach and LIME falls into the perturbation-based approach.

#### 2.7.1. Local Interpretable Model-Agnostic Explanations (LIMEs)

LIME [37,38,39] provides a local explanation by replacing a complex model locally with simpler models. The LIME optimization problem can be understood by introducing the necessary elements explained below.

For classification, $f\left(x\right)$ is the probability that x belongs to a certain class and f is the complex model being explained; G is the surrogate model used to approximate f in the vicinity of x. $\pi_{x}$ is the local neighborhood of x. $L(f,g,\pi_{x})$ indicates a measure of how the unfaithful g approximates f in a neighborhood defined by $\pi_{x}$. $\Omega \left(g\right)$ determines the complexity of the model g. The assumption made here is that complexity is inversely related to explaining ability. The explanation produced by LIME at a local point x can be determined using

(10) $$\xi \left(x\right)=\underset{g\in G}{\arg\min}\;L\left(f,g,\pi_{x}\right)+\Omega \left(g\right)$$

The objective is to assure interpretability and local fidelity while minimizing $L(f,g,\pi_{x})$ and maintaining less complexity $\Omega \left(g\right)$ to be understandable by humans. In summary, through the loss function $\xi \left(x\right)$ we can find a simple model g∈G where the model fidelity is maximized in the neighborhood $\pi_{x}$, keeping the interpretability as simple as possible.

The main aim is to minimize the local loss $L(f,g,\pi_{x})$ without making any assumptions about f, since explanations about the prediction by the function f should be model-agnostic. Thus, a combination of instances weighted by $\pi_{x}$ is generated in order to learn the local behavior of f as the inputs vary, and thus an approximation of $L(f,g,\pi_{x})$ is carried out. LIME creates new instances individually by changing each feature of input x from a normal distribution inferred from the training set. Given this dataset Z of perturbed samples with the associated labels, we optimize $\xi \left(x\right)$ to obtain a proper explanation about the stroke prediction.

#### 2.7.2. Shapley Additive Explanations (SHAP)

This is a model-agnostic approach (a concept from game theory). Shapley values determine the marginal contribution of every feature to the model’s output individually. But the disadvantage with SHAP is that it is resource intensive to compute, since it requires the assessment of many permutations [40,41]. The Shapley value is defined as

(11) $$\phi_{i}\left(v\right)=\sum_{S\subseteq N\backslash \left\{i\right\}}\frac{\left|S\right|!\left(\left|N\right|-\left|S\right|-1\right)!}{\left|N\right|!}\left(v\left(S\cup \left\{i\right\}\right)-v\left(S\right)\right)$$

Here, $v\left(S\cup \left\{i\right\}\right)-v\left(S\right)$ captures the marginal contributions of member i, $\left|S\right|!$ is the ways in which the set S could have been formed before adding i. $\left(\left|N\right|-\left|S\right|-1\right)!$ is the different ways the remaining players could be added and $\left|N\right|!$ is the number of combinations that can be formed with the coalition. The last three terms assign to the marginal contribution a specific weight accounting for all the different sequences in which the total coalition can be formed. The idea is that small and large subsets are more important; thus, they obtain a higher weight. For subsets with many players, we can learn about this player’s total effect (main effect plus feature interactions). For small subsets, we have isolated players and we can directly observe their contribution to the payout. Contrarily, if a coalition consists of half the players, we learn little about an individual feature’s contribution, as there are many possible coalitions with half of the players.

## 3. Results

The investigation for predicting brain strokes using four distinct machine-learning algorithms is presented in this part. Several quantitative criteria were used in the evaluation of these techniques, and they are discussed below:

### 3.1. Quantitative Evaluation

The confusion matrix for stroke prediction can be described as per Table 13.

Based on the above four parameters, various quantitative evaluations can be used to assess the performance of ML algorithms for stroke prediction. These evaluation metrics are accuracy (the proportion of accurate predictions made by the model relative to the total number of forecasts), precision (the percentage of accurate positive predictions out of all occasions when positive predictions were made), and recall (the proportion of genuine positive predictions made by the model out of the total number of real positive cases, also known as sensitivity or the true positive rate).

Log loss (cross-entropy) is also used for evaluation, which provides a more precise assessment of the model’s performance by penalizing inaccurate and unsure predictions calculated using

(12) $$\log\mathrm{loss}=-\frac{1}{N}\sum_{i=1}^{N}y_{i}\log\left(p\left(y_{i}\right)\right)+\left(1-y_{i}\right)\log\left(1-p\left(y_{i}\right)\right)$$

Here, $y_{i}$ is an actual label and $p\left(y_{i}\right)$ is the probability of the predicted labels. The performance of these is also graphically depicted by the receiver operating characteristic (ROC) curve.

Under different categorization criteria, it shows the true positive rate, $TPR=\frac{TP}{TP+FN}$ vs. the false positive rate, $FPR=\frac{FP}{FP+TN}$. The ROC curve sheds light on the trade-off between the model’s true positive rate and false positive rate, assisting in the selection of the ideal classification threshold. The ROC curve’s performance is summed up by the area under the curve (AUC). It measures how well the model can discriminate between various classes at all conceivable classification levels. AUC values closer to 1 show stronger discrimination between positive and negative cases, indicating better performance.

Three boosting algorithms, GB, XGB, and ADB, are applied in stroke prediction. Hyperparameter tuning is performed using a grid search. The hyperparameters used for GB and XGB are a learning rate of 0.1, a number of estimators of 100, and a maximum depth of 3; for ADB, the learning rate is 1.2 and the number of estimators is 5000. K-fold cross-validation is used to split the dataset into K = 5 folds to ensure that each fold is used as a validation set once while the remaining folds are used for training to provide robust evaluation of the model’s performance across different data subsets. The comparative analysis of accuracy obtained using these three boosting algorithms is shown in Table 14.

It can be observed from Table 14 that the training accuracy obtained using GB is 86.41% and the testing accuracy is 84.97%, which shows that GB performs reasonably well on both training and testing data. AdaBoost provides a training accuracy of 95.89% and a testing accuracy of 91.93%, which is higher than GB, indicating a better fit to the training data. The training accuracy obtained using XGB is 96.97% and the testing accuracy is 92.13%, which indicates that XGB demonstrates the highest training accuracy among the three algorithms, suggesting a very good fit to the training data. It also has the highest testing accuracy, indicating a better generalization performance compared to the other two algorithms.

**Figure 7:**
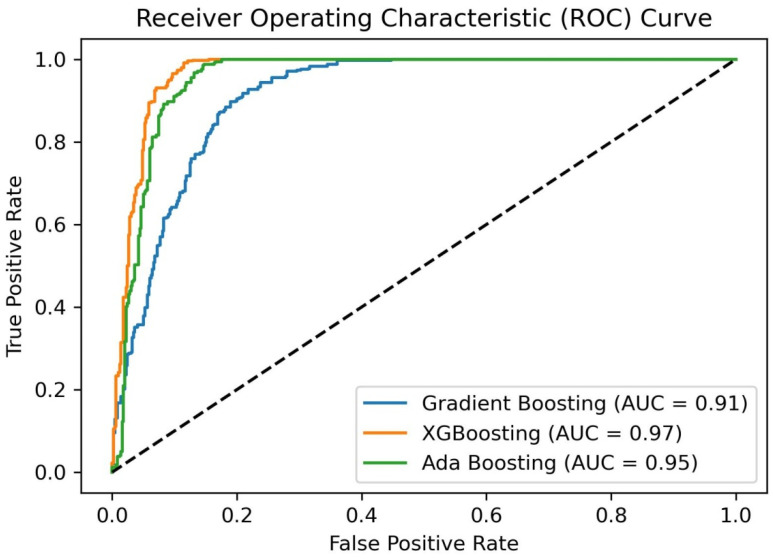
ROC and AUC values obtained using three ML algorithms for stroke predictions.

The ROC for the three ML algorithms is shown in Figure 7. It is observed that all three classifiers beat random chance (diagonal line) with high AUC ROC scores. XGB came out on top with nearly perfect discrimination. An AUC of 0.97 for XGB indicates that there is a 97% chance that the model will rank a randomly chosen positive instance higher than a randomly chosen negative instance. AdaBoost followed closely after, with a slightly lower AUC ROC (0.95) indicating a 95% chance that the model will rank a randomly chosen positive instance higher than a randomly chosen negative instance. Gradient boosting gives an acceptable AUC ROC of 0.91. The higher AUC of 0.97 for XGB indicates the better discriminative power of the model in distinguishing between the positive and negative classes.

**Figure 8:**
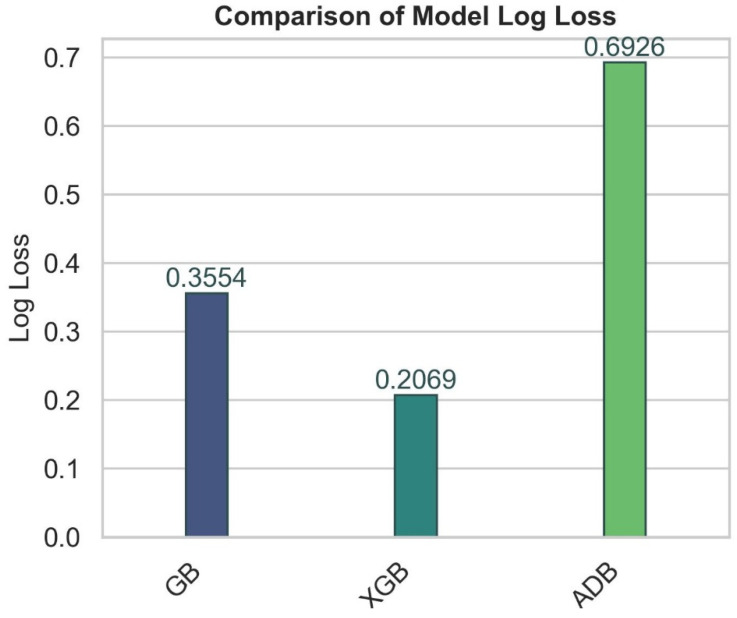
Model log loss obtained using GB, XGB, and ADB.

The performance of these models using log loss is shown in Figure 8.

**Figure 9:**
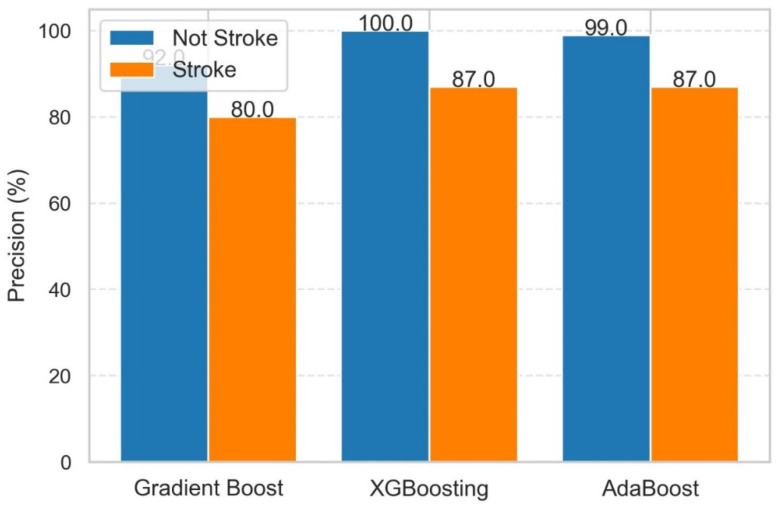
Precision in classifying stroke and non-stroke patients using GB, XGB, and ADB.

It can be observed from Figure 8 that XGB had the lowest log loss (0.206897), suggesting better probabilistic predictions than gradient boosting (0.355430) and AdaBoost (0.692624). The significant difference in log loss values demonstrates the capacity of XGB to generate more precise and accurate probability estimates for the specific challenge. The performance of these three models in terms of precision in classifying stroke and non-stroke patients is shown in Figure 9.

It can be observed from Figure 9 that all models were highly accurate in categorizing non-stroke patients (Class 0), with XGB attaining a perfect score (100%). Stroke cases (Class 1) were a difficulty, with accuracy ratings ranging from 80% to 87%. If we discuss class-specific performance, then the following classes can be described.

Non-Stroke (Class 0): XGB led with 100% accuracy, followed by AdaBoost (99%) and gradient boosting (92%).

Stroke (Class 1): All models had reduced accuracy, with XGBoost leading with 87%, followed by AdaBoost and gradient boosting with 87% and 80%, respectively.

**Figure 10:**
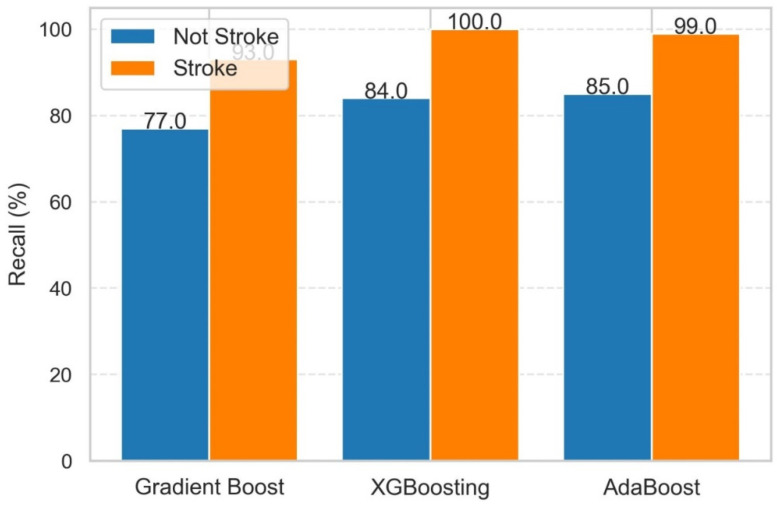
Recall in classifying stroke and non-stroke patients using GB, XGB, and ADB.

Thus, overall XGB outperforms precision for non-stroke instances, indicating great reliability in detecting genuine negatives. AdaBoost virtually equaled XGB for non-stroke instances and matched its precision for stroke cases, making it a viable option. Gradient boosting also appears to be helpful in non-stroke scenarios, despite its reduced accuracy in stroke scenarios. The performance of these three models in terms of recall in classifying stroke and non-stroke patients is shown in Figure 10.

It can be observed from Figure 10 that all models performed well in detecting stroke patients (Class 1), with XGB having an excellent recall of 100%. Non-stroke cases (Class 0) presented a difficulty, with recall rates ranging from 77% to 85%. If we consider class-specific performance, then the following classes can be described.

Stroke (Class 1): XGB showed the highest recall (100%), followed by AdaBoost (99%) and gradient boosting (93%).

Non-Stroke (Class 0): Recall scores were lower, with AdaBoost leading with 85%, XGB at 84%, and gradient boosting at 77%.

As a result, we are able to conclude that XGB outperforms in recall for stroke patients, assuring great sensitivity in detecting true positives. While beneficial in non-stroke patients, there is potential for improvement. ADB also almost equaled XGB in stroke instances while outperforming it in non-stroke cases, making it a viable option. Gradient boosting is also helpful in stroke patients; however, its reduced recall in non-stroke instances raises the possibility of missed detections.

**Figure 11:**
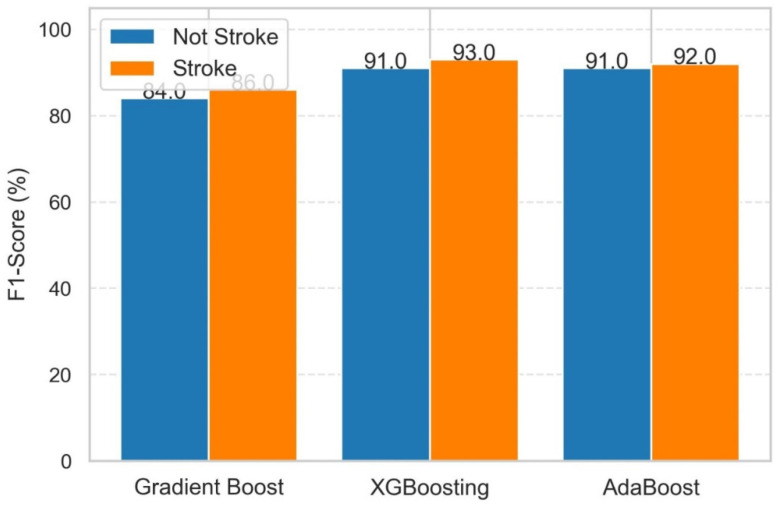
F1-score in classifying stroke and non-stroke patients using GB, XGB, and ADB.

The performance of these three models in terms of F1-score in classifying stroke and non-stroke patients is shown in Figure 11. All three models had excellent F1-scores, indicating a good balance of precision and recall in identifying stroke and non-stroke patients. XGBoost and AdaBoost routinely outperformed gradient boosting. If we talk about class-specific performance, then the following classes can be described.

Stroke (Class 1): XGB had the greatest F1-score (93%), closely followed by AdaBoost (92%), and gradient boosting (86%).

Non-Stroke (Class 0): XGB and AdaBoost matched for an F1-score of 91%, surpassing gradient boosting (84%).

As a result, XGB resulted as the overall best model, having the greatest F1-scores across both classes. From the quantitative evaluation, it is observed that XGB performs well as compared to ADB and GB. Hence, an explanation for stroke prediction is provided for XGB using LIME and SHAP.

### 3.2. Explanation Using SHAP and LIME

In this section, the explanation of the models’ output using SHAP and LIME is explained.

#### 3.2.1. SHAP Analysis

**Figure 12:**
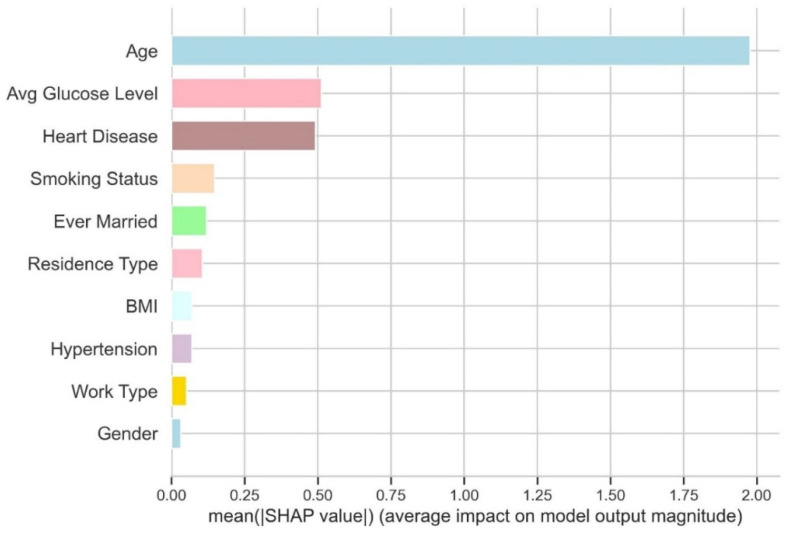
SHAP values for features contributing stroke.

The SHAP analysis is performed on the Extreme Gradient Boosting classifier. The SHAP values are calculated for the fitted model of the XGB classifier. Figure 12 shows the SHAP values for each feature contributing to strokes.

The features are ranks according to their average SHAP values, with the most important features at the top and the least essential at the bottom. This helps to understand how each attribute affects the model’s predictions. It can be observed from Figure 12 that each attribute has an equal influence on the categorization of stroke and non-stroke cases. The top four factors with the highest predictive potential are age, average glucose level, BMI, and smoking status. However, ever being married, residence type, gender, hypertension, work type, and heart disease make less of an impact than the previous four characteristics. This strategy can offer a more detailed summary of each feature’s effect on a single result (label). In the following example, SHAP values are used to represent the SHAP values for instances classed as having a stroke.

**Figure 13:**
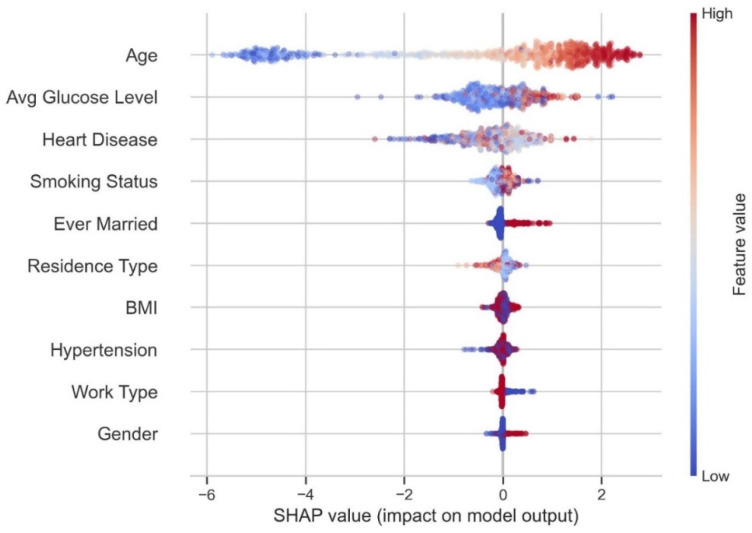
Impact of SHAP values on predicting stroke.

Figure 13 shows the impact of SHAP values on predicting strokes. The *y*-axis shows the characteristics sorted by their average absolute SHAP value. The *x*-axis indicates the SHAP values. Positive values for a particular feature move the model’s prediction closer to the label under consideration (label = 1). In contrast, negative values push towards the opposite class (label = 0). Individuals who are ageing are more likely to suffer from strokes. However, the model appears unsure about the diagnosis for younger individuals. Similarly, a high average glucose level (red dots) level increases the likelihood of being diagnosed with a stroke (positive result), whereas a low glucose level reduces the likelihood of being diagnosed.

#### 3.2.2. Local Interpretable Model-Agnostic Explanations (LIMEs) Analysis

Instead of offering a comprehensive explanation of the model over the whole dataset, LIME concentrates on explaining the model’s prediction for specific occurrences.

**Figure 14:**
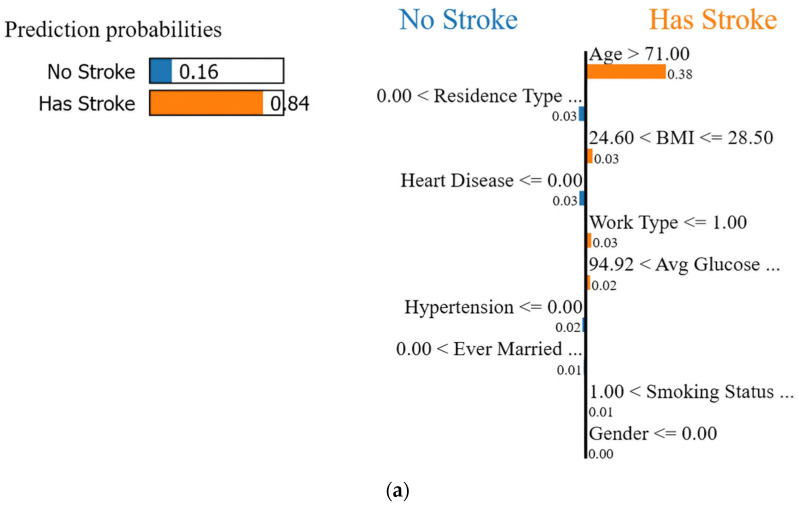

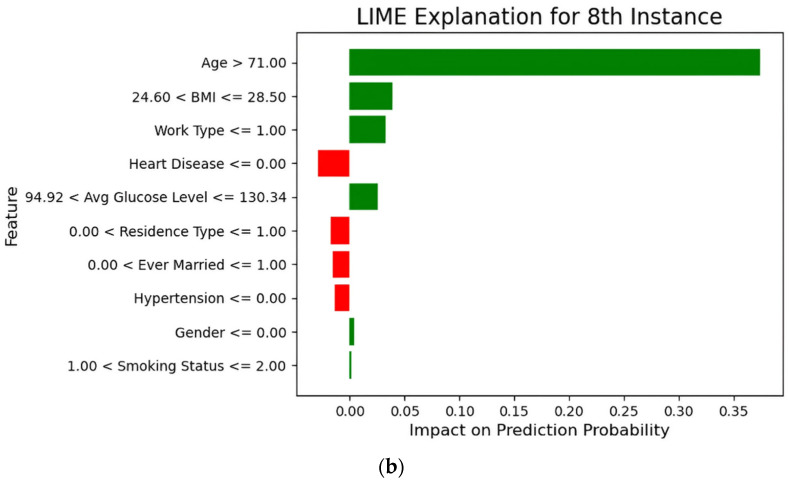
(**a**) LIME explanation for prediction of having a stroke for the 8th instance; (**b**) Range of feature values.

The LIME explanation for the eighth instance in the test data using XGB is demonstrated using Figure 14 and Table 9. As per the feature importance shown in Figure 14, it is observed that the eighth patient is predicted with an 84% chance of having a stroke. The major contributing parameters are age (greater than 71), body mass index (greater than 24.60), work type greater than or equal to 1 (indicating occupations such as government-employed, private-employed, and self-employed have chances of a stroke), average glucose level (greater than or equal to 94.92 and less than or equal to 130.34), gender (greater than or equal to zero indicating male or female, respectively), and smoking status (greater than or equal to 2 indicating a non-smoker).

Table 15 shows the exact values for the 8th instance which are responsible for predicting 84% chances of having a stroke. The values shown in the orange color contribute to strokes. These are the age of person being 79, body mass index being 27.20, the person being a non-smoker, female, and self-employed, and an average glucose level of 114.77.

**Figure 15:**
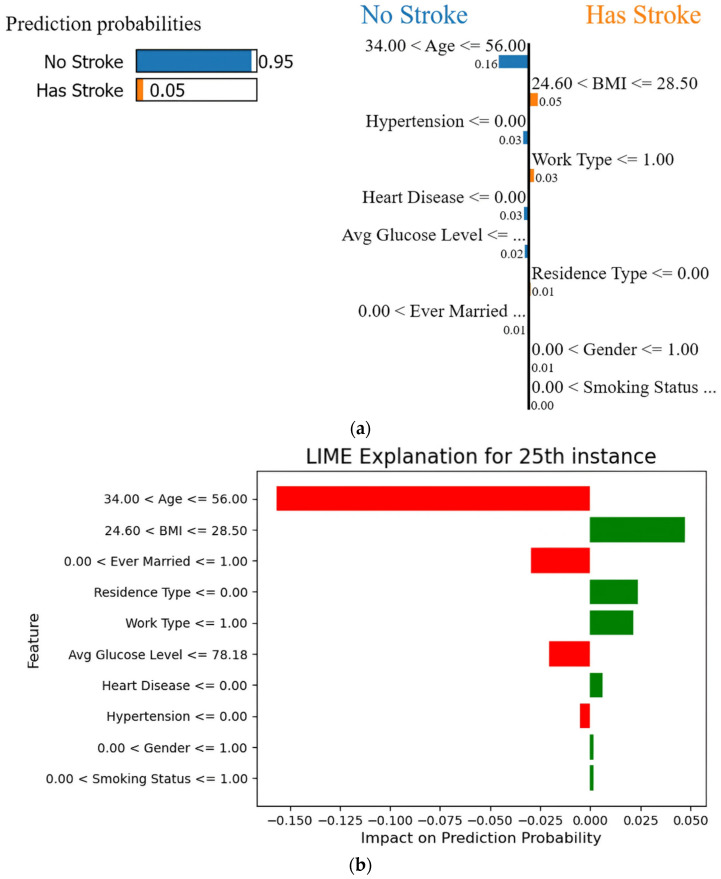
(**a**) LIME explanation for prediction of not having a stroke for the 25th instance; (**b**) Range of feature values.

In Figure 15, the 25th instance of LIME is examined, focusing on the stroke class to understand the impact of the features on stroke prediction. In this instance, it is observed that the prediction probability for no stroke is high at 95%. The major contributing features to this prediction include age (greater than 34 and less than or equal to 56), hypertension (less than or equal to zero, indicating no hypertension), average glucose level (less than or equal to 78.18), and ever being married (being married).

Table 16 shows the exact values for the 25th instance which are responsible for predicting 95% chances of not having a stroke. The values shown in the blue color are contributing factors for no stroke. The patient is a 38-year-old married person with no heart diseases and hypertension with an average glucose level of 77.35.

## 4. Discussion

The early detection of a brain stroke can significantly reduce the risk of severe complications and mortality with timely intervention. In this paper, ensemble-based boosting machine-learning algorithms are utilized to predict stroke risk. We proposed random sampling to addressed the issue of data imbalance, conducted a comprehensive analysis of features contributing stroke, and used an explainable model for interpretation to ensure transparency in decision-making. This section provides a discussion of the key findings, their implications, and the potential impact on clinical practice.

### 4.1. Contribution

- The random sampling method was used to balance the dataset to ensure that the model learned from both the minority (stroke cases) and majority (non-stroke cases) classes. The results demonstrate that the proposed sampling approach significantly enhanced the model performance in terms of precision, recall, and F1-score for both the classes.
- Feature analysis was conducted to investigate the impact of demographic features such as age and gender; physiological metrics like blood pressure, body mass index (BMI), and glucose levels; medical history elements, including a history of hypertension, heart disease, and diabetes; and lifestyle factors such as smoking habits and physical activity on stroke prediction.
- Explainable AI techniques such as LIME and SHAP were utilized to generate local explanations as well as for global interpretation of the results. It is observed that age is a strong predictor of stroke, and other features like glucose levels, BMI, heart disease, and work type also played a significant role, suggesting that a multifaceted approach is necessary for accurate stroke prediction.
- Optimized Boosting Algorithms: Gradient boosting (GB), XGBoost (XGB), and AdaBoost (ADB) were used to develop the stroke prediction model. The results show that XGBoost outperformed the other models with higher accuracy, precision, and recall.

### 4.2. Implications for Clinical Practice

The proposed method using random sampling, extensive feature analysis, and explainable AI offers significant potential for clinical applications. The proposed random sampling approach ensures that the model can identify stroke and non-stroke cases appropriately. The exhaustive feature analysis helps medical professionals to prioritize risk factors. Most importantly, the integration of LIME and SHAP ensures that the model’s predictions are transparent and interpretable. The explainability provided by LIME and SHAP assists healthcare providers in making informed decisions based on individual patient risk profiles.

### 4.3. Comparison with Existing Work

The comparison between different machine-learning models for stroke prediction reveals the dominance of the proposed algorithm over existing approaches in terms of accuracy and interpretability. The comparative analysis is shown in Table 17.

Logistic regression, random forest, XGBoost, KNN, SVM, and MLP in [42] achieved the best accuracy of 73.52%, with SHAP being the primary explainability tool. In contrast, a more diverse set of models, decision trees, random forests, logistic regression, SVM (linear and sigmoidal), KNN, AdaBoost, CatBoost, XGBoost, LGBM, and the stacking models of [43], achieved a significantly higher accuracy of 96%. These models leverage advanced ensemble techniques and boosting algorithms, which improve performance by reducing bias and variance. Multiple explainability methods like LIME, SHAP, Anchor, ELI5, and QLattice enhance interpretability, making the decision-making process transparent. The accuracy of the artificial neural network (ANN) model used in [44] was slightly less than the previous model at 95%. The proposed algorithm stands out with the highest accuracy of 96.97%. These boosting procedures have the same trait of enhancing effectiveness by addressing the difficult parts as objects of relative concentration which is very useful in medical prediction tasks. The increase in accuracy achieved in this new model as compared to the previous models exemplifies the improved generalization of the model across the particular dataset owing to the procedure of sequentially fixing the mistakes. In addition, both LIME and SHAP provide satisfactory construal of the manner in which the model’s behavior enables both advanced technology and high interpretation of the application.

## 5. Conclusions and Future Work

### 5.1. Conclusions

This paper proposed a systematic way of predicting stroke-patient survival using machine learning, assessing stroke prognosis by considering a wide range of factors such as demographic attributes, medical history, lifestyle elements, and physiological metrics. To provide robust model training and improve predictive models’ generalizability, an efficient random sampling approach was used. The model was tested with three algorithms: GB, AdaBoost, and XGB. The training accuracy obtained using GB was 86.41% and testing accuracy was 84.97%, which shows GB performs reasonably well on both training and testing data. AdaBoost provided a training accuracy of 95.89% and a testing accuracy of 91.93%, which is higher than GB indicating a better fit to the training data. The training accuracy obtained using XGB was 96.97% and the testing accuracy was 92.13% which indicates that XGB demonstrates the highest training accuracy among the three algorithms, suggesting a very good fit to the training data. It also had the highest testing accuracy, indicating a better generalization performance compared to the other two algorithms. Furthermore, the use of explainable AI approaches like LIME and SHAP enables transparent and interpretable model predictions. These strategies provide healthcare practitioners with useful insights into the underlying mechanisms that drive stroke prediction by explaining the contributions of specific characteristics to the predicted result. Overall, our work aims to give actionable insights that might help healthcare providers to develop personalized treatment plans for stroke patients. We want to improve the precision and efficacy of stroke care protocols by leveraging machine learning and explainable AI, resulting in better patient outcomes and quality of life.

### 5.2. Future Work

Although this research has demonstrated promising results on the Kaggle dataset for stroke prediction, future work should involve testing the model on multi-center datasets, which include data from various demographics, geographies, and healthcare systems, and longitudinal data that capture patient health metrics over a period of time. This will ensure that the model is robust and applicable to a wider range of patients and enhance the model’s predictive accuracy by identifying patterns of risk factor progression. Integrating the model into real-time clinical decision support systems would allow healthcare professionals to use the model’s predictions alongside traditional diagnostic tools, ultimately leading to more informed and timely interventions. Also, personalizing stroke prediction by incorporating genetic data or lifestyle modification interventions would allow for better risk assessment and prevention planning for individual patients.

## Figures and Tables

**Table 1:** Gender-wise distribution of stroke data.

Total Samples	Gender	Having Stroke (Yes/No)	
		Yes	No
4981	Women Patients (2074)	108	1966
	Male Patients (2907)	140	2767
	4981	248	4733

**Table 2:** Features with numerical values.

Sr. No.	Features	Minimum Value	Maximum Value	Mean Value	Average Value for Stroke	Average Value for Not Stroke
1.	Age	8	82	43	68	42
2.	Glucose Level	55.1	271.7	105.4	132.2	104.6
3.	BMI	14.0	48.9	28.5	30.2	28.4

**Table 3:** Features with categorical values.

Sr. No.	Parameter or Feature	Value or Attribute
1	Gender	0: Female 1: Male
2	Hypertension	0: No 1: Yes
3	Heart Disease	0: No 1: Yes
4	Ever Married	0: No 1: Yes
5	Work Type	0: Private 1: Self-employed 2: Government job 3: Never worked
6	Residence Type	0: Urban 1: Rural
7	Smoking Status	0: Unknown 1: Former 2: Never 3: Smokes

**Table 4:** Relation of hypertension on the outcome of stroke/not stroke.

Feature	Status	Count	Stroke	Not Stroke
Hypertension	Yes	479	66 (13.78%)	413 (86.22%)
	No	4502	182 (4.05%)	4320 (95.95%)

**Table 5:** Relation of heart disease on the outcome of stroke/not stroke.

Feature	Status	Count	Stroke	Not Stroke
Heart Disease	Yes	275	47 (17.09%)	228 (82.9%)
	No	4706	201 (4.2%)	4505 (95.72)

**Table 6:** Relation of marital status on the outcome of stroke/not stroke.

Feature	Status	Count	Stroke	Not Stroke
Ever Married	Yes	3280	219 (6.68%)	3061 (93.32%)
	No	1701	29 (1.70%)	1672 (98.3%)

**Table 7:** Relation of work type on the outcome of stroke/not stroke.

Feature	Status	Count	Stroke	Not Stroke
Work Type	Government Job	673	2 (0.3%)	671 (99.7%)
	Private Job	2860	148 (5.17%)	2712 (94.83%)
	Self-Employed	804	65 (8.08%)	739 (91.92%)
	Children	644	33 (5.12%)	611 (94.88%)

**Table 8:** Relation residence type on the outcome of stroke/not stroke.

Feature	Status	Count	Stroke	Not Stroke
Residence Type	Rural	2532	135 (5.33%)	2397 (94.67%)
	Urban	2449	113 (4.61%)	2336 (95.84%)

**Table 9:** Relation smoking status on the outcome of stroke/not stroke.

Feature	Status	Count	Stroke	Not Stroke
Smoking Status	Unknown	1500	47 (3.13%)	1453 (96.87%)
	Never	1838	89 (4.84%)	1749 (95.16%)
	Former Smoker	867	70 (8.07%)	797 (91.93%)
	Smoker	776	42 (5.41%)	734 (94.59%)

**Table 10:** Correlation values of the features affecting the chances of stroke.

Sr. No	Feature	Correlation Value with Stroke
1	Age	0.246
2	Heart Diseases	0.134
3	Average Glucose Level	0.133
4	Hypertension	0.131
5	Ever Married	0.108
6	Work Type	0.062
7	Gender	−0.008
8	Smoking Status	0.054
9	Body Mass Index	0.056
10	Residence Type	−0.016

**Table 11:** Dataset distribution after random downsampling.

Sampling Rate	Having Stroke (Yes/No)	Count
Downsampling by 50% on not-stroke samples	No	2480
100% of stroke samples	Yes	248
	Total	2728

**Table 12:** Dataset distribution after random upsampling.

Sampling Rate	Having Stroke (Yes/No)	Count
100% of not-stroke samples	No	2480
Upsampling by 10% on stroke samples	Yes	2480
	Total	4960

**Table 13:** Confusion matrix for stroke prediction.

	Predicted Class		
Actual Class		**Stroke**	**Not Stroke**
	Stroke	True Positive (TP): Patient with a stroke is predicted as having the possibility of a stroke	False Negative (FN): Patient with a stroke is mistakenly predicted as having the possibility of no stroke
	Not Stroke	False Positive (FP): Patient with no stroke is mistakenly predicted as having the possibility of a stroke	True Negative (TN): Patient with no stroke is predicted as having the possibility of no stroke

**Table 14:** The comparative analysis of accuracy (training and testing) using boosting algorithms.

Evaluation Metric	Machine-Learning Algorithms		
	Gradient Boosting	AdaBoost	XGBoost
Training Accuracy	86.41%	95.89%	96.97%
Testing Accuracy	84.97%	91.93%	92.13%

**Table 15:** Feature values of the 8th instance for prediction of having a stroke.

Age	79.00
Residence Type	1.00
BMI	27.20
Heart Disease	0.00
Work Type	1.00
Avg Glucose Level	114.77
Hypertension	0.00
Ever Married	1.00
Smoking Status	2.00
Gender	0.00

**Table 16:** Feature values of the 25th instance for prediction of not having a stroke.

Age	38.00
BMI	27.70
Hypertension	0.00
Work Type	1.00
Heart Disease	0.00
Avg Glucose Level	77.35
Residence Type	0.00
Ever Married	1.00
Gender	1.00
Smoking Status	1.00

**Table 17:** Comparison between existing models with our proposed methodology.

Sr. No.	Reference	Classifier	Best Accuracy	Explainable AI
1	[42]	Logistic regression, random forest, XGBoost, KNN, SVM, and MLP	73.52%	SHAP
2	[43]	Decision trees, random forests, logistic regression, SVM (Linear, sigmoidal), KNN, AdaBoost, CatBoost, LGBM, XGBoost, and stacking models	96%	LIME, Qlattice, SHAP, ELI5, Anchor
3	[44]	ANN	95%	LIME
4	Proposed Algorithm	Gradient boosting, XGBoost, AdaBoost	96.97%	LIME and SHAP

## Data Availability

The data presented in this study are openly available in Kaggle at https://www.kaggle.com/datasets/fedesoriano/stroke-prediction-dataset (accessed on 1 September 2024).
